# Irritable bowel syndrome and risk of colorectal cancer: a Danish nationwide cohort study

**DOI:** 10.1038/bjc.2011.65

**Published:** 2011-02-22

**Authors:** M Nørgaard, D K Farkas, L Pedersen, R Erichsen, Z D de la Cour, H Gregersen, H T Sørensen

**Affiliations:** 1 Department of Clinical Epidemiology, Clinical Institute, Aarhus University Hospital, Sdr. Skovvej 15, DK-9000, Aalborg, Denmark; 2 Mech-Sense, Aalborg Hospital, Aalborg, Denmark; 3 Sino-Danish Center for Education and Research, Beijing, China

**Keywords:** irritable bowel syndrome, colon cancer, risk, epidemiology

## Abstract

**Background:**

Little is known about the risk of colorectal cancer among patients with irritable bowel syndrome (IBS).

**Methods:**

We conducted a nationwide cohort study using data from the Danish National Registry of Patients and the Danish Cancer Registry from 1977 to 2008. We included patients with a first-time hospital contact for IBS and followed them for colorectal cancer. We estimated the expected number of cancers by applying national rates and we computed standardised incidence ratios (SIRs) by comparing the observed number of colorectal cancers with the expected number. We stratified the SIRs according to age, gender, and time of follow-up.

**Results:**

Among 57 851 IBS patients, we identified 407 cases of colon cancer during a combined follow-up of 506 930 years (SIR, 1.14 (95% confidence interval (CI): 1.03–1.25) and 115 cases of rectal cancer, corresponding to a SIR of 0.67 (95% CI: 0.52–0.85). In the first 3 months after an IBS diagnosis, the SIR was 8.42 (95% CI: 6.48–10.75) for colon cancer and 4.81 (95% CI: 2.85–7.60) for rectal cancer. Thereafter, the SIRs declined and 4–10 years after an IBS diagnosis, the SIRs for both colon and rectal cancer remained below 0.95.

**Conclusion:**

We found a decreased risk of colorectal cancer in the period 1–10 years after an IBS diagnosis. However, in the first 3 months after an IBS diagnosis, the risk of colon cancer was more than eight-fold increased and the risk of rectal cancer was five-fold increased. These increased risks are likely to be explained by diagnostic confusion because of overlapping symptomatology.

Irritable bowel syndrome (IBS) is the most common functional disorder of the gastrointestinal tract, characterised by abdominal pain, discomfort, and changes in bowel habits in the absence of known structural or biochemical abnormalities (Thompson *et al*, 2000). The prevalence of IBS in western countries ranges between 5–26%, depending on the criteria used to define the disease (Drossman *et al*, 1997; Hillila and Farkkila, 2004; Yale *et al*, 2008) and it is associated with repeated medical care visits and high costs (Sandler *et al*, 2002). Although a primary concern of IBS patients is their potential risk of developing colorectal cancer (Rey and Talley, 2009), few studies have evaluated the long-term course of IBS (Svendsen *et al*, 1985; Harvey *et al*, 1987; Owens *et al*, 1995; Garcia Rodriguez *et al*, 2000). In a follow-up study of 2956 IBS patients and 20 000 randomly sampled persons from the United Kingdom General Practitioners Database, with a mean follow-up time of 36 months, the relative risk (RR) of colorectal cancer was 5.8 (95% confidence interval (CI): 3.0–11.3) (Garcia Rodriguez *et al*, 2000). However, after excluding the first year of follow-up, the authors found that the RR was close to that of the general population (although no estimates were presented), arguing against a causal association.

As IBS is such a common condition, any associated increased risk of colorectal cancer is a public health concern. We therefore conducted a large nationwide follow-up study to examine the long-term risk of colon cancer among patients with a hospital diagnosis of IBS.

## Patients and methods

We conducted this nationwide study in Denmark within its population of 5.4 million habitants in 1977–2008. All Danish residents have a civil registration number, which is a unique personal identifier assigned at birth or upon immigration. This number is included in all public registries and allows unambiguous linkage between registries. The National Health Service provides tax-supported health care for all inhabitants of Denmark, guaranteeing free access to hospitals and general practitioners.

We identified all patients with a hospital contact (either admission or contact with a hospital-based outpatient clinic) for IBS using the Danish National Registry of Patients (DNRP), which has maintained records on all hospital admissions in Denmark since 1977. Visits to hospital-based outpatient clinics have been included since 1995. DNRP data includes civil registration number and up to 20 diagnoses, classified according to the *International Classification of Diseases*, eighth revision (ICD-8) until the end of 1993 and tenth revision (ICD-10) thereafter. We used the following codes to identify IBS: 564.19 in ICD-8, and K 58.0 and K 58.9 in ICD-10 and we defined date of diagnosis as the date of the first recorded hospital contact in the DNRP. To examine how colonoscopic surveillance affects long-term risk of colorectal cancer, we also identified patients who underwent colonoscopy or flexible sigmoidoscopy within 3 months before or after their first recorded IBS diagnosis, based on Nordic Classification of Surgical Procedures codes KUJF32-45. These codes have been included in the DNRP since the end of 1995.

Linkage to the Danish Cancer Registry allowed us to identify cancers diagnosed in patients with IBS. This is a population-based nationwide registry containing data on incident cases of cancer in Denmark since 1943 (Storm *et al*, 1997). Data recorded for each individual include civil registration number, method of cancer verification, stage, and residence on the date of cancer diagnosis. Diagnoses in the Cancer Registry have been coded according to ICD-10 since 2004. During the 1978–2003 period, all data were initially coded according to ICD-7; subsequently they were recoded using the ICD-10 classification. We used ICD-10 codes C18-C19 to identify cases of colon cancer and C20 to identify cases of rectal cancer. Patients with a cancer diagnosis before the date of their first IBS diagnosis were not included.

The civil registration number encodes age and gender. We computed age at the time of IBS diagnosis and categorised it into seven groups (<20 years, 20–39 years, 40–49 years, 50–59 years, 60–69 years, 70–79 years, and 80+ years).

### Statistical analyses

Follow-up started on the date of the first IBS-related hospital contact and continued until the date of a colorectal cancer diagnosis, the date of another cancer diagnosis, the date of death, the date of migration, or 31 December 2008, whichever came first. To compute standardised incidence ratios (SIRs) as a measure of relative risk, we estimated the expected number of cancers in our IBS population by applying rates from the Danish Cancer Registry (standardised on age, gender, and calendar time in 1-year intervals) to person-years of observation. We then compared the observed number of cases to the expected number and calculated a 95% CI, assuming that the observed number of cancers in a specific category followed a Poisson distribution. We computed SIRs for colorectal cancer overall and for colon and rectal cancer separately for the following follow-up periods: 0–91 days, 92–365 days, 366 days to 3 years, >3–5 years, >5–10 years, and >10 years. We stratified the analyses according to gender and age group. As colonoscopy enables detection and removal of precancerous lesions, we expected the risk of colorectal cancer to be reduced by this procedure (Brenner *et al*, 2010). We therefore conducted an analysis restricted to IBS patients with a recorded colonoscopy or flexible sigmoidoscopy. Since the DNRP was established in 1977, our estimates could be biased because of left-truncation of the data; we therefore conducted a subanalysis based on patients with a first IBS diagnosis in 1980–2008 and no IBS diagnosis in 1977–1979. We also conducted separate analysis for the two periods 1977–1994 and 1995–2008. We used SAS, version 9.1 (SAS Inc., Cary, NC, USA) for all analyses.

## Results

We identified 57 851 patients (39 998 women and 17 853 men) with a first-time IBS-related hospital contact. Of these, 44% were diagnosed as inpatients and 56% were diagnosed during a hospital-based outpatient visit. Median age at diagnosis was 47 years (interquartile range: 32–62 years).

During a combined follow-up of 506 930 years, we observed 522 cases of colorectal cancer with 530 expected, corresponding to a SIR of 0.99 (95% CI: 0.90–1.07). Within 3 months of IBS diagnosis, the SIR for colorectal cancer was 7.23 (95% CI: 5.75–8.97). In the period, 4 months to 1 year after IBS diagnosis, the SIR was 1.41 (95% CI: 1.04–1.87). The SIRs decreased to 0.89 (95% CI: 0.70–1.11) for the second and third years of follow up, 0.79 (95% CI: 0.61–1.02) for the fourth and fifth years of follow-up, 0.74 (95% CI: 0.60–0.89) for the sixth to the 10 years of follow up, and 0.83 (95% CI: 0.71–0.98) for those with more than 10 years of follow-up.

Among the 522 cancers, we identified 407 cases of colon cancer, including rectosigmoid cancer. This yielded a SIR of 1.14 (95% CI: 1.03–1.25) (Table 1). The SIR was 1.00 (95% CI: 0.81–1.21) for men and 1.19 (95% CI: 1.06–1.34) for women. Within 3 months of IBS diagnosis, the SIR for colon cancer was 8.42 (95% CI: 6.48–10.75). In the second and third years of follow-up, the SIR for colon cancer decreased to 1.08 (95% CI: 0.83–1.39). In the fourth and following years after IBS diagnosis, we consistently observed SIRs below 0.95 (Table 1).

We also identified 115 cases of rectal cancer among IBS patients during the study period, corresponding to a SIR of 0.67 (95% CI: 0.52–0.85) (Table 2). Within 3 months of IBS diagnosis, the SIR for rectal cancer was 4.81 (95% CI: 2.85–7.60), and in the second and third years of follow-up, the SIR for rectal cancer decreased to 0.50 (95% CI: 0.27–0.83), and after more than 10 years the SIR was 0.61 (95% CI: 0.42–0.85).

We identified 19 150 IBS patients diagnosed after 1995 who had undergone colonoscopy or flexible sigmoidoscopy within 3 months before or after the date of IBS diagnosis. Overall, the SIR of colon cancer in this group was 1.03 (95% CI: 0.78–1.34). However, after stratifying by follow-up period, we found that an increased risk of colon cancer was confined to the first 3 months following IBS diagnosis (SIR, 10.77 (95% CI: 7.03–15.78)) (Table 1). For rectal cancer, the SIR for the first 3 months following IBS diagnosis was 10.86 (95% CI: 5.78–18.58). After 4–12 months of follow-up, the SIR decreased to 1.13 (95% CI: 0.31–2.90). In the following years, the SIRs all suggested a protective effect of colonoscopy or flexible sigmoidoscopy (Table 2).

Table 3 shows observed and expected numbers of colon and rectal cancers, and corresponding SIRs for patients diagnosed in 1977–1994 and 1995–2008, separately. In 1995–2008, an increased risk of colon cancer was observed only in the first 3 months after IBS diagnosis. In 1977–1994, the risk was increased in the first 3 years after IBS diagnosis. For rectal cancer, the increased risk was only observed in the period 1995–2008 and the risk was confined to the first 3 months.

When we analysed data based on patients diagnosed with IBS in 1980–2008, we found only minor differences in our estimates (data not shown).

## Discussion

In this cohort study of nearly 60 000 IBS patients, we found no evidence of increased risk of colorectal cancer in the period starting 1 year after diagnosis. However, in the first 3 months after an IBS diagnosis, we found a more than eight-fold increased rate of colon cancer and a nearly five-fold increased rate of rectal cancer. In the following years, there was a lower risk of both colon and rectal cancer. If IBS was a cause of colorectal cancer, we would have expected the risk to increase with length of follow-up, because of these cancers’ long latency period. If, alternatively, IBS and colorectal cancer had common risk factors, a continued excess risk over time also would be expected. Our findings thus suggest that symptoms of colorectal cancer may initially be misinterpreted as IBS and then the associated clinical examinations subsequently lead to the diagnosis of colorectal cancer. In IBS patients without an early diagnosis of colorectal cancer, the risk remained lower than expected for at least 10 years. For rectal cancer, we even observed an overall 30% decreased risk in patients diagnosed with IBS, suggesting that rectal cancer is ruled out before an IBS diagnosis is given. We did not have information on colonoscopy for all our IBS patients, but when we restricted our analysis to those with a known colonoscopy or flexible sigmoidoscopy at the time of IBS diagnosis, the decreased long-term risk was even clearer.

Our findings are consistent with a study based on the UK General Practice Research Database (Garcia Rodriguez *et al*, 2000), which also found a nearly six-fold increased risk of colorectal cancer mainly confined to the first year after an IBS diagnosis. Our longer follow-up and larger sample size allowed us to extend these findings by showing a compensatory decreased risk in the second and following years after an IBS diagnosis. Although IBS is diagnosed according to symptom-based diagnostic criteria, use of routine sigmoidoscopy has been recommended by some experts (Camilleri and Prather, 1992; American Gastroenterological Association, 1997). In a multicenter randomised trial, Atkin *et al* (2010) demonstrated that once-only flexible sigmoidoscopy (during which polyps were removed) was associated with an IRR of colorectal cancer of 0.77 (95% CI: 0.70–0.84) during a median follow up of 11.2 years, compared with controls who did not undergo sigmoidoscopy. Brenner *et al* (2010) showed that the prevalence of left-sided colorectal neoplasms was clearly reduced within a 10-year period after colonoscopy; we found a similar effect in IBS patients.

Access to the Danish Cancer Registry allowed us to conduct a cohort study in a population-based setting with virtually complete follow-up of cancer cases. However, our data on IBS were based on diagnoses registered in the DNRP and it is known that these are not entirely accurate. Moreover, IBS has been defined differently over time, changing the clinical characteristics used to diagnose the disease. Thus, use of the Rome criteria results in a considerably lower prevalence of IBS compared with use of the Manning criteria (Boyce *et al*, 2006). Such diagnostic differences may explain the slightly higher risk of colon cancer among patients diagnosed in the first part of our study period. Since the DNRP was established in 1977, patients diagnosed with IBS in the first part of our study period may have been prevalent cases and not newly diagnosed cases. Changing our study period to 1980–2008 did not, however, change the results substantially – so any bias caused by left-truncated data is probably minor. Our use of routine data also may be a strength, as the study itself did not affect the process of diagnosing IBS. At the same time, IBS and colorectal cancer share symptoms, and the first symptoms of colorectal cancer may easily be confused with IBS. The increased risks of both colon and rectal cancers in the first year after the IBS diagnosis are thus likely to be explained by an incorrect IBS diagnosis (Garcia Rodriguez *et al*, 2000).

The restriction of our study to IBS patients diagnosed in a hospital contact may have produced some bias. Nevertheless, IBS patients without any hospital contacts should not be at higher risk of developing colon cancer than patients included in our study. In contrast, patients referred for evaluation in a hospital setting are likely to have a higher risk of colorectal cancer than those without hospital contacts if the referral reflects a higher level of concern about colorectal cancer. We therefore expect that such bias caused us to overestimate the risk. Restriction to patients diagnosed in 1995–2008 after inclusion of outpatient visits in the DNRP did not change our conclusions.

In conclusion, our study does not support the hypothesis that IBS is associated with an elevated risk of colorectal cancer. The increased risk of colorectal cancer in the first year after the IBS diagnosis is likely to be because of diagnostic confusion because of overlapping symptomatology between the two diseases.

## Figures and Tables

**Table 1:** Observed and expected numbers of colon cancers, including rectosigmoid cancers, and corresponding standardised incidence ratios in patients with irritable bowel syndrome overall and by period of follow-up, gender, age at diagnosis, and calendar period of diagnosis

	**All 57 851 IBS patients**			**19 150 IBS patients with known colonoscopy^a^**		
	**Observed cancers, *N***	**Expected cancers, *N***	**SIR (95% CI)**	**Observed cancers, *N***	**Expected cancers, *N***	**SIR (95% CI)**
Total	407	358.6	1.14 (1.03–1.25)	56	54.3	1.03 (0.78–1.34)
						
*Follow-up*						
⩽3 months	64	7.6	8.42 (6.48–10.75)	26	2.4	10.77 (7.03–15.78)
4–12 months	34	22.8	1.49 (1.03–2.08)	3	7.1	0.42 (0.09–1.24)
2nd and 3rd year	62	57.4	1.08 (0.83–1.39)	9	16.6	0.54 (0.25–1.03)
4th and 5th year	41	50.8	0.81 (0.58–1.10)	7	12.5	0.56 (0.22–1.15)
6th to 10th year	89	95.3	0.93 (0.75–1.15)	11	14.1	0.78 (0.39–1.39)
>10 years	117	124.7	0.94 (0.78–1.12)	0	1.6	—
						
*Gender*						
Women	302	253.1	1.19 (1.06–1.34)	34	35.0	0.97 (0.67–1.36)
Men	105	105.5	1.00 (0.81–1.21)	22	19.3	1.14 (0.71–1.73)
						
*Age*						
<20 years	0	0.3	—	0		
20–39 years	25	16.5	1.52 (0.98–2.24)	5	0.9	5.47 (1.77–12.75)
40–49 years	43	36.7	1.17 (0.85–1.58)	1	3.2	0.31 (0.01–1.73)
50–59 years	88	78.5	1.12 (0.90–1.38)	12	10.7	1.12 (0.58–1.95)
60–69 years	113	107.5	1.05 (0.87–1.26)	13	17.4	0.75 (0.40–1.28)
70–79 years	96	91.3	1.05 (0.85–1.28)	18	16.3	1.10 (0.65–1.74)
⩾80 years	42	27.8	1.51 (1.09–2.04)	7	5.7	1.23 (0.49–2.53)

Abbreviations: IBS=irritable bowel syndrome; CI=confidence interval; SIR=standardised incidence ratio.

a Colonoscopy or flexible sigmoidoscopy.

**Table 2:** Observed and expected number of rectal cancers and corresponding standardised incidence ratios in patients with irritable bowel syndrome overall and according to period of follow-up, gender, age at diagnosis and calendar period of diagnosis

	**All 57 851 IBS patients**			**19 150 IBS patients with known colonoscopy^a^**		
	**Observed cancers, *N***	**Expected cancers, *N***	**SIR (95% CI)**	**Observed cancers, *N***	**Expected cancers, *N***	**SIR (95% CI)**
Total	115	171.0	0.67 (0.52–0.85)	27	27.3	0.99 (0.65–1.44)
						
*Follow-up*						
⩽3 months	18	3.7	4.81 (2.85–7.60)	13	1.2	10.86 (5.78–18.58)
4–12 months	14	11.2	1.25 (0.68–2.09)	4	3.5	1.13 (0.31–2.90)
2nd and 3rd year	14	28.2	0.50 (0.27–0.83)	3	8.3	0.36 (0.07–1.05)
4th and 5th year	19	24.7	0.77 (0.46–1.20)	4	6.3	0.63 (0.17–1.62)
6th to 10th year	15	45.6	0.33 (0.18–0.54)	3	7.1	0.42 (0.09–1.23)
>10 years	35	57.5	0.61 (0.42–0.85)	0	0.8	—
						
*Gender*						
Women	71	102.7	0.69 (0.54–0.87)	12	14.6	0.82 (0.43–1.44)
Men	44	68.3	0.64 (0.47–0.86)	15	12.8	1.17 (0.66–1.94)
						
*Age*						
<20 years	0	0.2	—	0	0	—
20–39 years	6	9.6	0.62 (0.23–1.36)	1	0.5	1.85 (0.05–10.32)
40–49 years	14	20.7	0.68 (0.37–1.13)	3	2.1	1.40 (0.29–4.08)
50–59 years	26	41.9	0.62 (0.40–0.91)	2	6.8	0.29 (0.04–1.06)
60–69 years	30	50.2	0.60 (0.40–0.85)	13	9.0	1.45 (0.77–2.48)
70–79 years	24	37.6	0.64 (0.41–0.95)	3	6.7	0.45 (0.09–1.31)
⩾80 years	15	10.9	1.38 (0.77–2.28)	5	2.2	2.30 (0.75–5.36)

Abbreviations: IBS=irritable bowel syndrome; CI=confidence interval; SIR=standardised incidence ratio.

a Colonoscopy or flexible sigmoidoscopy.

**Table 3:** Observed and expected numbers of colon and rectal cancers, and corresponding standardised incidence ratios in patients with irritable bowel syndrome diagnosed in 1977–1994 and 1995–2008

	**1977–1994**			**1995–2008**		
	**Observed cancers, *N***	**Expected cancers, *N***	**SIR (95% CI)**	**Observed cancers, *N***	**Expected cancers, *N***	**SIR (95% CI)**
*Colon cancer*						
Total	256	221.1	1.16 (1.02–1.31)	151	137.5	1.10 (0.93–1.29)
*Follow-up*						
⩽3 months	22	2.7	8.03 (5.03–12.16)	42	4.9	8.63 (6.22–11.67)
4–12 months	21	8.5	2.48 (1.53–3.79)	13	14.4	0.91 (0.48–1.55)
2nd and 3rd year	33	22.5	1.47 (1.01–2.06)	29	34.9	0.83 (0.56–1.19)
4th and 5th year	20	22.0	0.91 (0.56–1.40)	21	28.8	0.73 (0.45–1.12)
6th to 10th year	48	51.1	0.94 (0.69–1.25)	41	44.2	0.93 (0.67–1.26)
>10 years	112	114.3	0.98 (0.81–1.18)	5	10.4	0.48 (0.16–1.12)
						
*Rectal cancer*						
Total	60	103.3	0.58 (0.44–0.75)	55	67.7	0.81 (0.61–1.06)
*Follow-up*						
⩽3 months	1	1.4	0.72 (0.02–4.03)	17	2.4	7.20 (4.19–11.54)
4–12 months	5	4.3	1.18 (0.38–2.74)	9	7.0	1.29 (0.59–2.45)
2nd and 3rd year	7	11.1	0.63 (0.25–1.30)	7	17.0	0.41 (0.16–0.85)
4th and 5th year	7	10.6	0.66 (0.27–1.37)	12	14.1	0.85 (0.44–1.48)
6th to 10th year	9	23.7	0.38 (0.17–0.72)	6	21.9	0.27 (0.10–0.60)
>10 years	31	52.3	0.59 (0.40–0.84)	4	5.3	0.76 (0.21–1.95)

Abbreviations: CI=confidence interval; SIR=standardised incidence ratio.

## References

[bib1] American Gastroenterological Association (1997) American Gastroenterological Association medical position statement: irritable bowel syndrome. Gastroenterology 112: 2118–2119 9178708 10.1053/gast.1997.1122118

[bib2] Atkin WS, Edwards R, Kralj-Hans I, Wooldrage K, Hart AR, Northover JM, Parkin DM, Wardle J, Duffy SW, Cuzick J (2010) Once-only flexible sigmoidoscopy screening in prevention of colorectal cancer: a multicentre randomised controlled trial. Lancet 375: 1624–1633 20430429 10.1016/S0140-6736(10)60551-X

[bib3] Boyce PM, Talley NJ, Burke C, Koloski NA (2006) Epidemiology of the functional gastrointestinal disorders diagnosed according to Rome II criteria: an Australian population-based study. Intern Med J 36: 28–36 16409310 10.1111/j.1445-5994.2006.01006.x

[bib4] Brenner H, Hoffmeister M, Arndt V, Stegmaier C, Altenhofen L, Haug U (2010) Protection from right- and left-sided colorectal neoplasms after colonoscopy: population-based study. J Natl Cancer Inst 102: 89–95 20042716 10.1093/jnci/djp436

[bib5] Camilleri M, Prather CM (1992) The irritable bowel syndrome: mechanisms and a practical approach to management. Ann Intern Med 116: 1001–1008 1586089 10.7326/0003-4819-116-12-1001

[bib6] Drossman DA, Whitehead WE, Camilleri M (1997) Irritable bowel syndrome: a technical review for practice guideline development. Gastroenterology 112: 2120–2137 9178709 10.1053/gast.1997.v112.agast972120

[bib7] Garcia Rodriguez LA, Ruigomez A, Wallander MA, Johansson S, Olbe L (2000) Detection of colorectal tumor and inflammatory bowel disease during follow-up of patients with initial diagnosis of irritable bowel syndrome. Scand J Gastroenterol 35: 306–311 10766326 10.1080/003655200750024191

[bib8] Harvey RF, Mauad EC, Brown AM (1987) Prognosis in the irritable bowel syndrome: a 5-year prospective study. Lancet 1: 963–965 2882351 10.1016/s0140-6736(87)90304-7

[bib9] Hillila MT, Farkkila MA (2004) Prevalence of irritable bowel syndrome according to different diagnostic criteria in a non-selected adult population. Aliment Pharmacol Ther 20: 339–345 15274671 10.1111/j.1365-2036.2004.02034.x

[bib10] Owens DM, Nelson DK, Talley NJ (1995) The irritable bowel syndrome: long-term prognosis and the physician-patient interaction. Ann Intern Med 122: 107–112 7992984 10.7326/0003-4819-122-2-199501150-00005

[bib11] Rey E, Talley NJ (2009) Irritable bowel syndrome: novel views on the epidemiology and potential risk factors. Dig Liver Dis 41: 772–780 19665952 10.1016/j.dld.2009.07.005

[bib12] Sandler RS, Everhart JE, Donowitz M, Adams E, Cronin K, Goodman C, Gemmen E, Shah S, Avdic A, Rubin R (2002) The burden of selected digestive diseases in the United States. Gastroenterology 122: 1500–1511 11984534 10.1053/gast.2002.32978

[bib13] Storm HH, Michelsen EV, Clemmensen IH, Pihl J (1997) The Danish Cancer Registry--history, content, quality and use. Dan Med Bull 44: 535–539 9408738

[bib14] Svendsen JH, Munck LK, Andersen JR (1985) Irritable bowel syndrome--prognosis and diagnostic safety. A 5-year follow-up study. Scand J Gastroenterol 20: 415–418 4023607 10.3109/00365528509089673

[bib15] Thompson WG, Heaton KW, Smyth GT, Smyth C (2000) Irritable bowel syndrome in general practice: prevalence, characteristics, and referral. Gut 46: 78–82 10601059 10.1136/gut.46.1.78 PMC1727778

[bib16] Yale SH, Musana AK, Kieke A, Hayes J, Glurich I, Chyou PH (2008) Applying case definition criteria to irritable bowel syndrome. Clin Med Res 6: 9–16 18591372 10.3121/cmr.2008.788 PMC2442028

